# Sun Safe Partners Online: Pilot Randomized Controlled Clinical Trial

**DOI:** 10.2196/18037

**Published:** 2020-09-17

**Authors:** Sharon Manne, David Buller, Katie Devine, Carolyn Heckman, Sherry Pagoto, Sara Frederick, Anna Mitarotondo

**Affiliations:** 1 Rutgers Cancer Institute of New Jersey New Brunswick, NJ United States; 2 Klein Buendel, Inc Golden, CO United States; 3 University of Connecticut Storrs, CT United States

**Keywords:** sun protection, behavior intervention, online interventions, couples, skin cancer prevention, mobile phone

## Abstract

**Background:**

Harnessing supportive influences in close relationships is an innovative and potentially effective strategy to improve sun protection behaviors.

**Objective:**

This pilot randomized controlled clinical trial evaluates the feasibility and impact of Sun Safe Partners Online, a web-based, couples-focused intervention to improve sun protection behavior.

**Methods:**

A total of 75 couples reporting suboptimal levels of sun protection recruited from Facebook advertisements were randomized to receive a web-based intervention called Sun Safe Partners Online or a Generic Online Sun Safety Information intervention. Sun Safe Partners Online had 4 individual-focused modules and 4 couples-focused modules. Feasibility was assessed by study enrollment, engagement, follow-up survey completion, and intervention evaluation. Participants completed baseline and a 1-month postintervention survey assessing sun protection and exposure, along with individual and relationship attitudes about the importance of sun protection.

**Results:**

Using Facebook as a recruitment strategy resulted in rapid enrollment and higher acceptance than for the prior telephone and print trial. The follow-up survey completion was higher in the Generic Online condition (100%) than in the Sun Safe Partners Online condition (87.2%). Engagement in Sun Safe Partners Online was high, with more than two-thirds of participants completing all modules. Evaluations of Sun Safe Partners Online content and features as well as ease of navigation were excellent. Sun Safe Partners Online showed small effects on sun protection behaviors and sun exposure on weekends compared with the Generic Online intervention and moderate effect size increases in the Sun Safe Partners Online condition.

**Conclusions:**

This study uses a novel approach to facilitate engagement in sun protection by harnessing the influence of relationships among spouses and cohabiting partners. A couples-focused intervention may hold promise as a means to improve sun protection behaviors beyond interventions focused solely on individuals by leveraging the concern, collaboration, and support among intimate partners and addressing relationship-based barriers to sun protection.

**Trial Registration:**

ClinicalTrials.gov NCT04549675; https://clinicaltrials.gov/ct2/show/NCT04549675

## Introduction

Skin cancer is the most common cancer in the United States. An estimated 96,480 cases of invasive melanoma and 5.4 million cases of nonmelanoma skin cancer were diagnosed in 2019 [1] Melanoma is the fifth most common malignancy in both men and women [2]. The rate of new melanoma cases has been rising by 1.5% on average each year over the last 10 years [2]. The incidence and mortality rates of non-melanoma squamous cell skin cancer are also increasing. The number of deaths caused by squamous cell skin cancer may soon be comparable to melanoma-related deaths. The rising number and per person costs of treatment for skin cancer has increased the average national annual treatment costs of skin cancer, estimated at US $8.2 billion per year [3]. On the basis of these facts, the United States Surgeon General’s Call to Action to Prevent Skin Cancer [4] emphasized that skin cancer is a serious public health concern and suggested heightened skin cancer prevention efforts, including research, surveillance, and evaluation.

The primary risk factor for skin cancer is excess exposure to UV light, and the majority of skin cancers could be prevented if people consistently engaged in sun protection [5-7], The American Cancer Society [1] and the Skin Cancer Foundation [8] recommend minimizing exposure between daily peak hours for UV exposure, using sunscreen with a sun protection factor (SPF) of 30 or higher regularly and wearing protective clothing. Engagement in these recommendations is low. Up to 72% of US population do not use sunscreen regularly, wear protective clothing, or avoid the sun while outdoors [9,10]. Many studies have evaluated individual factors that contribute to sun protection behaviors, including demographic variables, objective risk factors, and attitudes and beliefs. For example, fewer perceived benefits of sun protection, more barriers to sun protection, and lower self-efficacy for using sun protection predict less sun protection [11-14].

The majority of sun protection interventions also focus on individuals. The potential role of the marital relationship as a motivator for sun protection is a less-studied, yet important, factor. Couples live together and typically engage in activities together. Thus, they share situations where UV exposure occurs (eg, sports events, vacations to sunny places), share sun protection equipment (eg, sunscreen bottles), and share environmental support for sun protection habits (eg, a car where sunglasses are stored). Overall, the high correlation between partners’ sun protection practices (*r*=0.5-0.6) indicates significant couple similarity with regard to sun protection [15]. In terms of marital relationship influences, couples who discuss sun protection and endorse its benefits for the other partner and their relationship are more likely to engage in sun protection [15]. The marital relationship is an important influence on sun protection, and harnessing constructive marital influences offers a promising method to improve sun protection. Although no studies have evaluated the mechanisms of marital influence on sun protection, some have examined general family influence in persons with a family history of melanoma. These studies suggest that greater family support for sun protection is associated with higher levels of sun protection, and that communication about skin cancer occurs within families, particularly between parents and their minor children [16,17]. Additionally, family-focused behavioral interventions have shown efficacy in promoting health-related behaviors, including physical activity and diet as well as sun protection habits [18-20].

When considering how marital relationships may influence health behavior, Lewis et al [21] proposed an integrative framework based on an interdependence theory and communal coping framework to explain how couples’ interactions may influence engagement in risk-reducing health behavior. This framework proposes that a strong interdependence in long-term, successful close relationships (ie, partners’ influence on one another’s behaviors and outcomes) transforms their motivations from doing what is in their self-interest (self-centered) to doing what is in the best interest of their relationship (relationship-centered). The transformation from self- to relationship-centered motivation occurs when partners ascribe health threats and subsequent health changes as having meaning for the relationship and/or their spouse. The model by Lewis et al [21-24] proposed 4 contributors to behavioral change: (1) predisposing factors of the couple (eg, individual perceptions of the health threat), (2) how much partners agree that health changes should be made together, (3) partners’ commitment to the relationship, and (4) demographic factors. When relationship-centered motivation develops, communal coping begins. Communal coping efforts consist of joint decision making (eg, discussing the change) and planning how to make the change. Communal coping efforts lead to engagement in health behavior change for both partners [21-24]. In our prior work [15], we found high couple concordance with sun protection practices (*r*=0.5) and support for the interdependence and communal coping framework. Couples who reported that they discussed sun protection and endorsed its benefits for their partner and their relationship were more likely to engage in sun protection. Taken together, this suggests that harnessing constructive relationship influences via behavioral interventions may be a promising method for improving sun protection.

In a prior study, we developed and tested a couple-focused print and telephone counseling intervention called Sun Safe Partners [25]. Content was guided by the interdependence and communal coping framework. It included the provision of mailed small media materials, a couple-focused telephone counseling call, and a mailed summary letter. Results from a small, nonrandomized trial showed that couples’ sun protection behaviors significantly increased after the intervention. We also observed increases in attitudes about the importance of one’s own engagement in sun protection for the partner, relationship, and partner-centered motivations to engage in better sun protection [25]. However, intervention uptake was low, and implementation was challenging for our enrollees; it was difficult to schedule couples for the 1-hour phone call, deliver the content, and create implementation plans to improve sun protection for both partners.

To address these challenges, we created Sun Safe Partners Online and utilized a social media recruitment strategy rather than a web-based panel strategy. The web-based intervention allowed couples to work through the content at convenient times and at their own pace without the need for an interventionist. In addition to standard individual-focused behavior change strategies such as goal setting and planning better sun protection, Sun Safe Partners Online content targeted couple-level influences by (1) raising awareness of the partner’s skin cancer risk, (2) identifying benefits of improving sun protection for the partner and relationship, (3) helping partners learn and practice constructive communication to foster one another’s sun protection, (4) identifying ways the partner can assist in working on sun protection behavioral goals, and (5) providing home assignments to help the couple discuss sun protection and ways to support each other’s goals. Furthermore, content was added to address the risks of sun exposure to children, assess a child’s risk factors, and set sun protection goals for the child for couples who have children in the home. We chose a social media advertisement recruitment strategy to examine whether this strategy resulted in better uptake than our prior work [25].

In this study, we report on the development, feasibility, and pilot testing of Sun Safe Partners Online. In a pilot and randomized feasibility trial, we compared Sun Safe Partners Online with a Generic Sun Safety Information-Only Online condition. The study had 2 aims. The first aim was to evaluate the feasibility and acceptability of Sun Safe Partners Online as compared with the Generic Online intervention. Feasibility was measured as enrollment, retention, and intervention use. Acceptability was assessed by a self-report evaluation of both interventions. We also compared our social media recruitment approach to the web-based panel approach utilized in our previous study [25]. The second aim was to assess the impact of Sun Safe Partners Online on the primary outcomes of sun protection and sun exposure and our intervention processes, which were individual and relationship attitudes and practices about sun protection. A 1-month, postbaseline follow-up survey was administered to examine the short-term impact of the intervention.

## Methods

### Development of Sun Safe Partners Online

Over a 10-month period, we worked with ITX Corporation to develop an interactive, online-mobile responsive (ie, can be accessed on a smartphone as well as a desk or laptop computer) web-based intervention. The web-based intervention focused on both the individual and the relationship with content divided into *My Stuff* (individual content) and *Our Stuff* (couple content). As shown in Table 1, we addressed key individual knowledge, attitudes, and behavior change constructs for sun protection as well as relationship content. Content from the original print and telephone intervention was used. We focused on individual factors drawn from Jackson and Aiken’s psychosocial model of sun protection [13]. Sun Safe Partners Online also targeted proven behavior change techniques, including goal setting, action planning, and reviewing behavioral goals.

**Table 1:** Individual- and couple-focused objectives, targeted constructs, and tasks for Sun Safe Partners Online.

Objectives, Targeted constructs		Key tasks in Sun Safe Partners
**Improve attitudes and skills for better sun protection**		
	Personal risk for skin cancer Sun protection benefits Sun protection barriers Improve confidence in sun protection practices Action planning and goal setting	Increase awareness of personal risk factors for skin cancer Provide information about recommended sun protection Assess current sun protection behaviors Improve awareness of benefits of sun protection Assess and address personal barriers to sun protection practices Provide education about sunscreen application, sunscreen, sunglasses, minimizing exposure, unintentional sun exposure, and the dangers of tanning Set sun protection behavioral goals, develop plans to address barriers to change
**Build relationship focus and support for sun protection**		
	Promote awareness of the benefits of sun protection for partner and relationship Promote acceptance of partner support and influence	Increase awareness of how the marital relationship can foster better health practices Identify the benefits to the partner and the relationship for engaging in better sun protection Increase awareness of partner’s skin cancer risk (phenotype and current sun protection) Increase willingness to accept influence from one’s partner
	Promote supportive relationship behaviors regarding sun protection and including partner in goals	Identify desired support for sun protection from the partner Understand how to provide constructive support to one’s partner Build the ability to give and accept partner’s influence for sun protection Increase understanding of constructive communication to foster better sun protection habits Include partner support for sun protection goals in goal-setting exercise

The program allowed couples to access the material while at home, but we did not convey the expectation that they would log in together and view the material at the same time. Rather, each partner had their own link and password to open the intervention. In the Our Stuff modules, we designed activities that provided information about the other partner (eg, feedback about the other partner’s skin cancer risk factors) as well as home assignments that ask couples to discuss topics covered in the modules and engage in setting joint goals and support each other’s behavior change goals. Some of the activities (eg, completing quizzes) would be difficult to complete at the same time.

**Figure 1:**
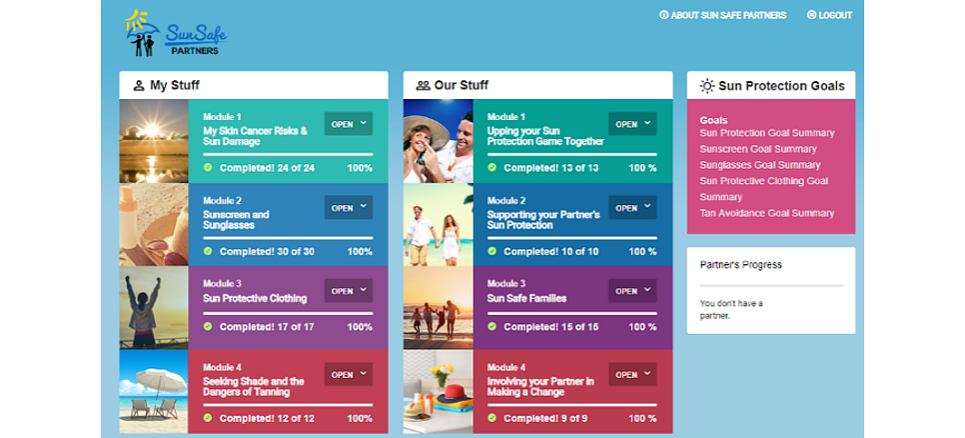
Sun Safe Partners’ landing page.

We developed 4 modules. Each *My Stuff* module included information displayed in colorful and engaging ways, tailored self-assessments (eg, Fitzpatrick skin type risk calculation, current sun protection, and sunscreen barriers), individual feedback (eg, participant’s Fitzpatrick skin type), goal-setting exercises (eg, select a goal, identify barriers, and develop strategies to address barriers), and downloadable PDF files (eg, daily sun exposure diary). In *Our Stuff* modules, we created novel approaches to build relationship support. Each module contained basic information (eg, benefits of improving your health for your partner and relationship, the importance of staying healthy for your spouse and relationship, and shared environments for sun protection), personalized assessments (eg, my partner’s risk factors for skin cancer, what my partner can do to help my sun protection, and how my partner can help with my sun protection goal), and couples home assignments (eg, share your sun protection goal with your partner). *Our Stuff* module 2 contained an animated video illustrating couples’ communication about sun protection and partner assistance in completing a sun protection goal. Sun Safe Partners Online included a separate sun protection goal summary module, where participants could review and update their goals for sun protection, sunscreen, sunglasses, sun-protective clothing, and tanning avoidance. Sun Safe Partner’s navigation page included a partner progress area, where the partner’s progress was displayed. Participants could *nudge* their partner to log into the website or complete content on the landing page. Table 2 contains a summary of the content and assignments for each module, and the landing page for Sun Safe Partners Online is shown in Figure 1. After the initial development, we sent Sun Safe Partners Online to 6 couples to review and provide input and comments on navigation and content. Their feedback was incorporated into the intervention. A key change was made to unlock modules. Initially, the team planned to unlock the 4 modules weekly. However, owing to participant feedback, all modules were unlocked so that participants could complete the modules at their own pace.

**Table 2:** Information on content of the Sun Safe Partners Online modules.

Modules	My Stuff	Our Stuff
1	My skin cancer risks and sun damage: Rationale for making changes as a couple Basic information about skin cancer and sun damage Assessment of skin cancer risk Additional risks (sunburn history, tanning) Ways to protect yourself from the sun Assessment of current sun protection practices Set and plan a sun protection goal	Upping your sun protection game together: Importance of spouse support for health behavior changes Health behavior changes made in the past that benefitted the partner and/or relationship Choose relationship benefits for improving sun protection Select way that partner can help with protection goal Homework: share sun protection goal with partner or discuss relationship benefits of better sun protection
2	Sunscreen and sunglasses: Homework review Sunscreen recommendations, UV-A and UV-B, what is sun protection factor, chemical versus physical sunscreens Set a sunscreen goal Sunglasses: Ask the expert, barriers to wearing sunglasses Set a sunglasses goal	Supporting your partner’s sun protection: Understanding your partner’s skin cancer risk and current sun protection How to support your partner improving his/her sun protection Homework: Discuss skin cancer risk factors and sun protection behaviors that partners have in common/do not have in common or share your sun protection goal and make a plan about how you can help one another
3	Sun protective clothing: Homework review Recommended types of clothing Unintentional sun exposure Barriers to wearing clothing and hats Set a sun protective clothing goal	Sun safe families (for couples who have children in the home): Phenotypic risk assessment of child who has worst sun protection Why children are at increased risk/guidelines Sun safety in the home and outside the home Assess child sun protection behaviors Assess parent barriers for child sun protection Setting a goal for child sun protection
4	Seeking shade and the dangers of tanning: Homework review Dangers of tanning How to protect your skin by seeking shade Rating current sun protection behaviors Set a shade or tanning goal	Involving your partner in making a change: Review of risk factors, sunscreen, sun glasses, protective clothing, and sun avoidance recommendations and how to involve your partner in the changes List benefits to partner, benefits to relationship, and what partner can do to help you make the change
Goal setting	Goals from each section are imported into this section. Participants can view, add, and/or modify goals	N/A^a^

^a^N/A: not applicable.

For the trial, consented participants were emailed a link to Sun Safe Partners Online. On the landing page, participants were instructed to complete home assignments before proceeding to the next module. As noted above, participants were instructed to move to the next module once the home assignments were completed.

### Generic Online Intervention

The team reviewed publicly online available skin cancer and sun protection information and selected the following 4 links to send to participants as the Generic Online comparison condition: (1) The Skin Cancer Foundation’s Skin Cancer Prevention guidelines [8], (2) the Centers for Disease Control and Prevention’s information on risk factors for skin cancer [7], (3) the American Academy of Dermatology’s information on how to select an appropriate sunscreen [26], and (4) The Skin Cancer Foundation’s information on sun protective clothing [27]. Participants were emailed a link to one of the resources each week for 4 weeks.

### Participants and Procedures

Facebook advertisements were created with Oxford Communications, an advertising company, to recruit couples for this study. The eligibility criteria were (1) both partners aged 18 to 75 years, (2) married or cohabiting with a significant other for at least one year, (3) partner #1 must be willing to provide contact information for partner #2, the (4) panel member (partner #1) and spouse responded with never, rarely, sometimes, or often to the question, “When outdoors in warm weather, how often do you protect your skin (by staying in the shade or covering your body with protective clothes or 30+ SPF sunscreen)?” (those who responded with *always* were excluded), (5) both partners had not been diagnosed with any type of skin cancer, and (6) both partners had an email account, internet access, and phone service (cell or landline).

Enrollment procedures were as follows: first, partner #1, the person who viewed the Facebook advertisement and indicated an interest in participating, clicked on the advertisement that took partner #1 to the eligibility survey. This survey included a consent to answer screening questions. If partner #1 was eligible, this person provided contact information for their cohabiting significant other (partner #2) and himself or herself. Next, Partner #2 was emailed a link to the eligibility survey. If both partners were eligible, then a member of the study team called the couple and spoke with both partners to confirm study eligibility. The team member confirmed study eligibility and sent an electronic link to the web-based consent and survey to eligible couples. Participants followed the link to acknowledge reading the web-based consent document before proceeding to the survey.

After both partners consented and completed the survey, couples were randomly assigned to either the Sun Safe Partners Online or the Generic Online condition. The cancer center’s biostatistician created the randomization scheme Individual assignments were stored in a locked Excel file that could only be accessed by the study’s project coordinator and accessed sequentially according to completion of the baseline assessment. Couples randomized to Sun Safe Partners Online were registered on the website and were provided a unique username and password. Participants were instructed to work at their own pace, but asked to do home assignments before logging into the next module. Home assignments were exercises completed with the other partner. Couples randomized to the Generic Online intervention were emailed the initial link, with 1 of 3 additional email links sent every 5 days. Participants were enrolled from May to August 2019, with participants recruited using 4 waves of advertisements. Follow-up surveys were completed between May and November 2019.

At baseline and the 1-month follow-up, participants completed surveys assessing sun protection, sun exposure, sun protection intentions, sun protection benefits, sunscreen and clothing barriers, and self-efficacy for sun protection as well as relationship benefits, motivation, and support. At the 1-month follow-up, a treatment acceptability measure was completed. Time spent in modules was downloaded from the Sun Safe Partners Online website. Participants were paid US $25 for the baseline and US $25 for the follow-up survey.

### Measures: Primary Outcomes

#### Sun Protection

The Sun Habits survey [28] asked participants to rate their frequency of 5 sun protection behaviors (sunscreen, hat, shirt with sleeves, long pants, and sunglasses) on warm sunny days (1=never to 5=always). Studies evaluating the validity of self-reported sun protection with weekly electronic diaries [29] and observational assessments of sun protection have reported good correspondence [30,31]. Alpha reliabilities ranged from .52 to .64.

#### Sun Exposure

The Sun Habits survey [28] asked participants to rate the duration of outdoor time during peak hours on weekends and weekdays over the past summer months, 1=30 min or less, and 8=more than 6 hours. Self-report measures of time outdoors have shown satisfactory agreement with observational and dosimeter methods [29].

### Measures: Intervention Processes

#### Individual Attitudes

Three items assessed the perceived risk of skin cancer (sample item: “If I don’t protect myself from the sun, I would feel vulnerable to getting skin cancer in my lifetime”) [15,32]. Items were rated on Likert-type response scales, ranging from 1 (strongly disagree) to 5 (strongly agree); alphas ranged from .91 to .92. Nine items assessed sun protection benefits (sample item: “Regularly wearing sunscreen when in the sun would reduce my chances of getting skin cancer”); alphas ranged from .85 to .87 [13,33]. Another 9 items measured sunscreen barriers (sample item: “For me, using sunscreen when I am outside on a warm sunny day is not part of my daily routine”); alphas ranged from .78 to .87 [13,34], both on 1 (strongly disagree) to 5 (strongly agree) Likert-type scales. Barriers to wearing sun-protective clothing [11] were measured with 7 Likert-type items. Sample item: “For me, wearing sun protective clothing when I am outside on a warm sunny day interferes with my work or leisure activities,” 1 (strongly disagree) to 5 (strongly agree); alphas ranged from .82 to .95. Finally, self-efficacy for sun protection [13,33] was assessed with 9 items on confidence in performing sun protection behaviors. Sample item: “Are you confident that you can use sunscreen on every part of your body that is not covered by clothing?” rated from 1 (not at all confident) to 5 (very confident); alpha was .83 at both time points.

#### Relationship Attitudes

Twelve items measured the relationship benefits of sun protection for one’s partner and relationship. Sample item: “I can think of reasons it would be beneficial for my relationship if I engage in sun protection,” 1 (strongly disagree) to 5 (strongly agree); alphas ranged from .93 to .94 [15]. Five items assessed relationship motivations, that is, the degree to which partners perceive it is important to engage in sun protection because it is important to the other partner. Sample item: “I wear sunglasses when I go outside because it is important to my spouse that I do so,” 1 (not at all true) to 5 (very true); alphas ranged from .80 to .87 [15]. Participants rated whether they engaged in 10 support behaviors for sun protection in the past month. Sample item: “Encouraged my spouse to apply sunscreen”; alphas ranged from .73 to .81 [25]. One item assessed the degree to which participants received support for sun protection from their spouse who supported their sun protection. Sample item: “How supportive is your partner of your sun protection practices?” (1=not at all supportive to 5=very supportive) [25].

#### Demographics

Age, sex, education, season of year enrolled, state residing in during childhood, relationship length, and phenotypic risk were measured at baseline. Phenotypic risk was measured using the Brief Cancer Risk Assessment scale [35]. Eight items assessed risk factors for skin cancer (sample item: “What is the color of your non-sun exposed skin?”)

#### Intervention Acceptability, Satisfaction, and Use

At the 1-month follow-up, participants in both conditions completed a 9-item scale about the intervention they received. Sample items: “How helpful were the materials?” 1 (not at all helpful) to 7 (extremely helpful); “I learned something new from the materials/Sun Safe Partners website,” “The information was easy to understand,” and “I feel the materials were prepared with me and my partner in mind,” 1 (strongly disagree) to 7 (strongly agree). Participants also rated how much of the materials they viewed. Sun Safe Partners: “How much of the Sun Safe Partners website did you review?”; Generic Online: “How much of the materials did you review?” 1 (just the overview) to 7 (viewed it many times) [25].

Sun Safe Partner Online participants completed a measure of ease of navigation, evaluation of content and features, and overall satisfaction. Twelve items assessed ease of navigation (sample items: “The Sun Safe Partners website was easy to use” and “The Sun Safe Partners website was user friendly”; rated on a 7-point Likert scale, 1=strongly disagree to 7=strongly agree). Eight items assessed features of the Sun Safe Partners program. Sample items: “What did you think about the goal-setting features throughout the program?” and “What did you think of the homework discussions with your partner?” (1=not at all helpful to 7=extremely helpful). Four items evaluated satisfaction. Sample item: “I am satisfied with the Sun Safe Partners website,” 1 (strongly disagree) to 7 (strongly agree). The Sun Safe Partners Online website tracked logins and time in modules (eg, both partners individually in each couple).

### Analytic Plan

For aim 1, in addition to basic descriptive information (eg, acceptance and survey completion rates), we compared the 2 study aims with regard to treatment evaluation. Both members of the couple participated in the study, hence the data were not independent. To handle this nonindependence, we used multilevel modeling treating dyad as the upper-level unit to compute tests of the intervention effect (Sun Safe Partners vs Generic Online). For aim 2, we adopted the same approach but in addition to the treatment condition, the fixed effect model also controlled for the person’s baseline score on the outcome. Note that because this is a pilot study, we report Cohen *d* effect sizes that were computed based on the *t* values and degrees of freedom for the condition effect from the multilevel models. Given the small data set and the goals of the pilot study, missing data were not imputed.

## Results

### Participants

The sociodemographic characteristics of the sample are shown in Table 3. The sample was 47.3% male and 52.3% female (there were several same-sex couples). Most (79.6%) participants were non-Hispanic White (9.5% Asian, 5.4% Black, and 3.4% Hispanic White), 88% had at least a high school certificate, the average age was 39.5 years (range 24 to 69 years), and the median relationship duration was 12 years (range 3 to 43 years). Nearly all participants (93.2%) had major medical insurance. Regarding sun exposure, 54.7% had experienced 3 or more blistering sunburns in their lifetime, and 42% had engaged in indoor tanning at least once in the past.

**Table 3:** Descriptive information for the study sample.

Variables		Sun Safe Partners Online		Generic Online intervention	
		Males	Females	Males	Females
Age (years), mean (SD)		40.9 (9.0)	38.2 (7.6)	41.0 (9.2)	38.1 (8.7)
**Race and ethnicity, n (%)**					
	Non-Hispanic White	27 (75.0)	31 (77.5)	27 (77.0)	31 (83.8)
	Non-Hispanic Black	2 (5.6)	2 (5.0)	1 (2.9)	1 (2.7)
	Hispanic White	4 (11.1)	1 (2.5)	1 (2.9)	1 (2.7)
	Asian	3 (8.3)	4 (10.0)	5 (14.3)	4 (10.8)
	Indigenous people	0 (0)	1 (2.5)	1 (2.9)	0 (0.0)
	Other	0 (0.0)	1 (2.5)	0 (0.0)	0 (0.0)
**Education, n (%)**					
	Less than high school	1 (2.8)	0 (0.0)	0 (0.0)	0 (0.0)
	High school	6 (16.7)	4 (10.0)	4 (11.4)	3 (8.1)
	Some college	9 (25.0)	11 (27.5)	7 (20.0)	6 (16.2)
	Bachelor’s degree	11 (30.5)	15 (37.5)	11 (31.4)	16 (43.2)
	Graduate degree	9 (25.0)	10 (25.0)	13 (37.2)	12 (32.5)
Relationship length (years), mean (SD)		13.8 (7.6)	12.9 (7.8)	13.0 (8.0)	12.5 (7.7)
Insurance status (yes), n (%)		33 (91.7)	36 (90.0)	33 (97.1)	35 (94.6)
**Childhood residence (sun exposure), n (%)**					
	Northern latitude	29 (80.6)	29 (72.5)	23 (65.7)	26 (70.3)
	Southern United States	1 (2.8)	0 (0.0)	0 (0.0)	1 (2.7)
	Hawaii or Tropics	6 (16.6)	9 (22.5)	11 (31.4)	8 (21.6)
	Unknown	0 (0.0)	2 (5.0)	1 (2.9)	2 (5.4)
**Phenotypic risks, n (%)**					
	Fair to very fair skin	23 (63.9)	31 (77.5)	20 (58.8)	24 (68.6)
	Blonde or red hair	8 (22.2)	10 (25.0)	4 (11.8)	9 (25.8)
	History of 6 or more sunburns	6 (16.7)	12 (30.0)	4 (11.7)	10 (28.6)
	More than 10 moles	2 (5.6)	2 (5.0)	0 (0.0)	1 (2.9)
	Many freckles	7 (19.4)	12 (30.0)	3 (8.8)	12 (34.3)
	Burn easily	17 (47.2)	28 (71.8)	19 (55.9)	22 (62.9)
	Ability to tan none or light	11 (30.6)	20 (50.0)	12 (35.3)	18 (51.4)

### Aim 1: Feasibility and Acceptability

#### Recruitment and Retention

**Figure 2:**
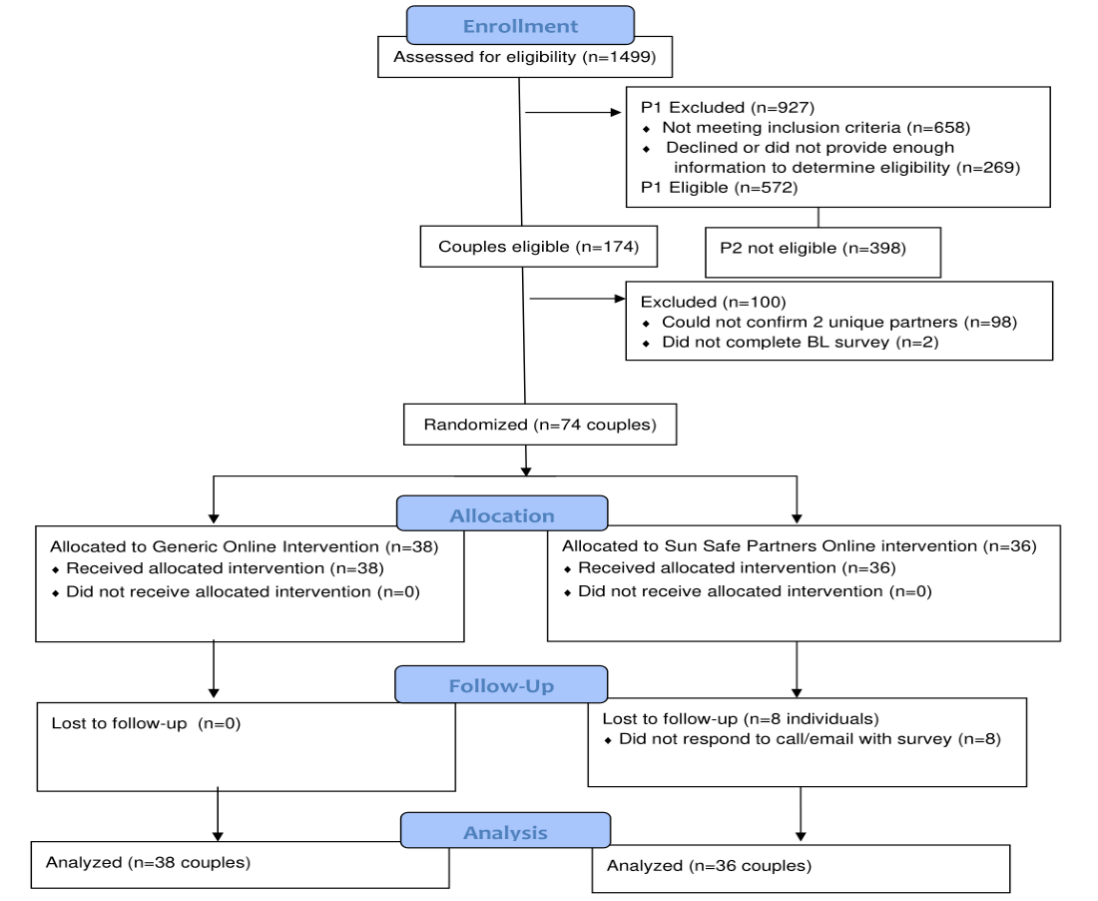
CONSORT diagram. BL: baseline; CONSORT: Consolidated Standards of Reporting Trials; P1: Patient #1; P2: Patient #2.

The recruitment and retention of participants is shown in the Consolidated Standards of Reporting Trials (CONSORT) diagram in Figure 2. From Facebook advertisements, 572 eligible partner #1s were identified, and links were sent by the project coordinator to partner #2 to determine eligibility. Of these 572 partners #1s, 398 partner #2s (69.5%) did not complete the eligibility screener or were ineligible, and 174 partner #2s (30.5%) were eligible. These 174 couples were contacted by phone, and 77 couples (44%) were reached and confirmed to be an actual couple. Of these 77 couples, 74 couples completed baseline surveys and were randomized to the Sun Safe Partners Online or the Generic Online intervention (n=36 couples/74 participants assigned to the Sun Safe Partners condition and 38 couples/74 participants assigned to the Generic Online condition). This yielded an acceptance rate of 42.5% (74/174 couples) of eligible couples.

Figure 2 shows the retention rate. The follow-up survey completion rate was 93.3%. However, the return rate was higher in the Generic Online condition (100%) than in the Sun Safe Partners Online condition (87.2%). Comparisons between participants who completed the follow-up with participants who did not complete the follow-up with regard to demographic characteristics, baseline individual and relationship attitudes, sun protection, and sun exposure, did not show significant differences in any of the variables.

#### Sun Safe Partners Online Engagement and Evaluation

Of the 78 participants randomized to Sun Safe Partners Online, a review of data collected from the website indicated that 72 (92.3%) logged into the first module. The percentage of participants who opened the first page of each module are as follows: *My Stuff*: module 1 (91.5%), module 2 (91.5%), module 3 (83.5%), and module 4 (78.2%); *Our Stuff*: module 1 (84.6%), module 2 (76.9%), module 3 (76.9%), and module 4 (75.6%). The total time spent in Sun Safe Partners Online ranged from 1.5 min to 189.2 min (median 67.5 min, mean 69.3 min, SD 47.7 min). Of the 72 participants randomized to the Generic Online intervention, 60 participants (83.3%) reported reviewing all materials at least once, and 6 reported that they glanced over it or viewed a few sections (7.3%).

Of the participants randomized to the Sun Safe Partners Online, who completed the follow-up survey, 95.5% reported completing homework discussions with their partner. Among participants reporting having a discussion with their partner, the most common home assignment topics that were discussed were: “Sharing my sun protection goal with my partner and how he or she can support me” (70.5%), “Making a plan about how you can help each other work on your sun protection goal”(62.8%), “Working together on building a sun safe home”(61.5%), “Discussing the benefits to your relationship, partner, and family of adopting better sun protection” (60.3%), and “Discussing skin cancer risk factors and sun protection habits that my partner and I have in common and do not have in common” (60.3%). Intervention acceptability is shown in Table 4.

Both interventions were evaluated positively. However, Sun Safe Partners Online had significantly higher ratings than the Generic Online intervention on helpfulness, learning something new, being valuable, being interesting, and being prepared with the couple in mind, making it easier to talk to the partner about better sun protection, and fostering an understanding of why it is helpful for the relationship and spouse to engage in better sun protection. Sun Safe Partners Online was also rated highly on ability to navigate it and its content. Videos, interactive quizzes, and homework assignments were rated positively. Positive aspects noted in open-ended questions were as follows: “interesting videos,” “like connection with my partner,” and “examples of how to talk to my husband about sun protection.”

**Table 4:** Feasibility and acceptability of Sun Safe Partners Online and Generic Online intervention.

Intervention acceptability			Sun Safe Partners (n=67), mean (SD)		Generic Online (n=72), mean (SD)		*t* test (*df*)		*P* value
**General characteristics**									
	Was helpful	6.10 (1.08)		5.60 (1.27)		2.01 (70)		.048	
	Contained valid information	6.55 (0.70)		6.39 (0.99)		1.06 (70)		.29	
	Learned something new	6.34 (1.04)		5.69 (1.33)		2.64 (70)		.01	
	Information was valuable to me	6.31 (0.93)		5.57 (1.37)		2.94 (68)		.005	
	Information was interesting	6.24 (1.00)		5.51 (1.34)		2.87 (68)		.005	
	Length of time to review it was sufficient	5.57 (1.56)		5.40 (1.50)		0.44 (70)		.66	
	Prepared with me and my partner in mind	6.30 (1.10)		5.32 (1.51)		3.80 (69)		<.001	
	Made it easier to talk to my partner about better sun protection	6.27 (1.25)		5.43 (1.42)		3.16 (70)		.002	
	Helped me understand why it was helpful for our relationship and why my spouse has to protect our skin from the sun	6.43 (1.06)		5.52 (1.33)		3.77 (70)		<.001	
**Sun Safe Partners Online navigation**									
	Easy to use	6.58 (0.68)		N/A^a^		N/A		N/A	
	Simple to use	6.56 (0.77)		N/A		N/A		N/A	
	User friendly	6.53 (0.75)		N/A		N/A		N/A	
	Required fewest steps possible to accomplish what I wanted to do	5.92 (1.33)		N/A		N/A		N/A	
	Flexible	6.29 (0.98)		N/A		N/A		N/A	
	Using it was effortless	6.09 (1.12)		N/A		N/A		N/A	
	Learned to use it quickly	6.52 (0.83)		N/A		N/A		N/A	
	Easy to remember how to use it	6.57 (0.72)		N/A		N/A		N/A	
	Easy to learn to use it	6.54 (0.84)		N/A		N/A		N/A	
	Quickly became skillful	6.43 (0.93)		N/A		N/A		N/A	
**Sun Safe Partners Online satisfaction**									
	Satisfied with it	6.45 (0.82)		N/A		N/A		N/A	
	Would recommend to a friend	6.22 (1.18)		N/A		N/A		N/A	
	Works the way I want it to	6.28 (0.93)		N/A		N/A		N/A	
	Feel the need to have it	5.70 (1.43)		N/A		N/A		N/A	
**Features of Sun Safe Partners**									
	Sun protection content	6.31 (1.00)		N/A		N/A		N/A	
	Videos	5.73 (1.58)		N/A		N/A		N/A	
	Home assignments for couple	5.93 (1.25)		N/A		N/A		N/A	
	Quizzes	6.30 (1.13)		N/A		N/A		N/A	
	Links between partner answers	6.17 (1.02)		N/A		N/A		N/A	
	Reminder to login	6.01 (1.28)		N/A		N/A		N/A	
	Goal-setting feature	5.76 (1.46)		N/A		N/A		N/A	

^a^N/A: not applicable.

### Aim 2: Impact of Sun Safe Partners Online Versus Generic Online Intervention on Outcomes

The descriptive statistics for baseline and follow-up as a function of condition as well as *t* tests testing the condition effect and Cohen *d* estimating the condition effect are presented in Table 5. As the levels of sun exposure on weekdays were quite low (33% reported 30 min or less on weekdays vs only 3% reported 30 min or less on weekends), we focused on weekend sun exposure as the sun exposure outcome.

**Table 5:** Comparisons of the Sun Safe Partners Online with the Generic Online intervention on outcomes and relationship and individual factors.

Outcomes^a^			Sun Safe Partner Online		Generic Online			Cohen *d*
			Baseline, mean (SD)	Follow-up, mean (SD)	Baseline, mean (SD)	Follow-up, mean (SD)		
**Primary outcomes**								
	Sun Protection Behaviors		2.84 (0.67)	3.19 (0.73)	2.79 (0.60)	2.96 (0.59)	0.36	
	Weekend sun exposure		3.58 (1.59)	2.84 (1.33)	3.54 (1.58)	2.93 (1.21)	0.08	
**Intervention processes**								
	**Relationship factors**							
		Relationship benefits	4.39 (0.66)	4.66 (0.52)	4.01 (0.71)	4.26 (0.65)	0.68	
		Relationship motivation	1.93 (0.72)	2.54 (0.96)	1.73 (0.72)	2.17 (0.87)	0.29	
		Support provided	4.33 (0.94)	4.59 (0.67)	4.06 (1.14)	4.58 (0.78)	0.18	
		Support received	4.45 (0.84)	4.47 (0.87)	3.94 (1.17)	4.31 (1.03)	0.18	
	**Individual factors**							
		Sun protection intentions	4.35 (1.13)	5.35 (1.09)	4.00 (1.03)	4.77 (1.04)	0.48	
		Perceived risk	4.27 (0.94)	4.47 (0.94)	4.01 (0.96)	4.30 (0.87)	0.01	
		Sun protection benefits	4.37 (0.74)	4.76 (0.53)	4.17 (0.69)	4.49 (0.55)	0.45	
		Sunscreen barriers	2.74 (0.73)	2.39 (0.87)	2.90 (0.69)	2.58 (0.83)	0.14	
		Clothing barriers	3.41 (1.12)	3.07 (1.24)	3.74 (0.97)	3.28 (1.15)	0.01	
		Sun protection efficacy	3.29 (0.88)	3.64 (0.83)	3.14 (0.87)	3.44 (0.83)	0.16	

^a^At baseline, Sun Safe Partners Online (n=76) and Generic Online (n=72). At follow-up, Sun Safe Partners Online (n=72). The *t* tests for follow-up differences as a function of condition were computed using multilevel modeling controlling for baseline score. Degrees of freedom for these tests ranged between 64 and 72 across the variables.

Sun Safe Partners Online showed small-to-moderate size effects on sun protection behaviors and sun exposure on weekends as compared with the Generic Online intervention. Sun Safe Partners Online also showed small-to-moderate size effects on relationship benefits and support provided to the partner. The small-to-moderate effect sizes for individual factors suggested that participants in Sun Safe Partners Online increased sun protection intentions and benefits.

## Discussion

Engagement in Sun Safe Partners Online was high, with the vast majority of participants using it, and more than two-third of participants completing all modules. Online delivery may have allowed couples to engage in the program at convenient times and places, at their own pace, and when they were together.

Using Facebook as a recruitment strategy resulted in rapid recruitment. Higher acceptance rates were observed (43%) relative to our prior telephone and print trial (22.4%) [25], which used a Qualtrics online panel recruitment. Compared with a Qualtrics panel, Facebook users may also have a preference for online interventions, which could explain the higher acceptance rate. Facebook is also a networking platform that may have yielded participants who placed a higher premium on a relationship-based intervention. It would be informative to see if samples recruited through general internet advertising or offline means would be as engaged with the Sun Safe Partners Online intervention as the current sample derived from Facebook. Follow-up survey completion was also high, although the return rate was lower in the Sun Safe Partners Online condition than in the Generic Online condition. Perhaps the participants from Facebook expected to be engaged for a shorter period of time, and thus, some of them felt they had devoted enough time to the study when completing the Sun Safe Partner Online intervention. Still, follow-up in the Sun Safe Partners Online condition was still high (87%), reducing concerns that loss to follow-up would create substantial selection biases.

Compared with the Generic Online intervention, Sun Safe Partners Online showed small magnitude increases in sun protection behaviors and sun exposure on weekends. However, it should be noted that there were increases in sun protection and reductions in sun exposure in the Generic Online intervention. This was encouraging and suggests that the provision of basic sun protection and skin cancer risk information to participants may motivate some increase in sun protection behaviors. However, these changes must be interpreted with caution. Owing to a very small sample, we avoided significance testing, and the effect size may be imprecise, as noted in the published advice on interpreting pilot studies [36,37]. The results hold promise due to Sun Safe Partners Online’s differential impact on relationship factors. Although both interventions showed increases in participants’ ability to view sun protection from a relational perspective, there were moderate-sized increases in the Sun Safe Partners Online condition and small magnitude effects for Sun Safe Partners Online on relationship motivations and support for the spouse’s sun protection. The results support the framework by Lewis et al [38]. These findings are corroborated by the fact that participants reported supportive behaviors to help their partner adopt sun protection behaviors. In terms of individual factors, there were medium effect sizes in favor of Sun Safe Partners on sun protection benefits and intentions, but there were no differential effects on sunscreen barriers, barriers to wearing sun-protective clothing, and perceived risk. Overall, the pattern of findings implies that Sun Safe Partners Online had an impact on the relationship constructs as intended. However, a fully powered randomized trial is needed to provide inferential tests on the effectiveness of Sun Safe Partners Online [36,37].

A comparison with our previous noncontrolled clinical trial in which Sun Safe Partners involved a tailored counseling call as well as print materials [25] illustrates some important points about Sun Safe Partners Online. In our prior study, we reported a larger effect size for sun protection behaviors (Cohen *d*=1.29) at the 6-month follow-up than the effect size for sun protection behaviors in this trial (Cohen *d*=.36) at the 1-month follow-up. However, these 2 effect sizes are not directly comparable in the sense that the effect size from this study compares baseline to follow-up differences between the intervention and control groups, and the effect size from the previous study compared only differences between baseline and follow-up for the intervention group (ie, there was no control). We recomputed both effect sizes using data from only those individuals in the intervention group who completed both waves of data collection. On the basis of these data, we again found that the current web-based study produced a much smaller overtime effect size (Cohen *d*=.43 vs Cohen *d*=1.41). There are several possible explanations for this difference. First, the mean baseline score on sun protection behaviors for the study was considerably higher (mean 2.88, SD 0.70) than in the previous study (mean 2.47, SD 0.47). Thus, these differences may reflect sampling. A second potential explanation for this finding is that the prior intervention was more intensive, and because all individuals participated in the counseling call, the intervention *dose* was more consistent.

Although both interventions were positively evaluated, Sun Safe Partners Online was rated as more helpful, valuable, and interesting than the Generic Online intervention. Participants felt that it was prepared for the couple, it was viewed as promoting the ability to talk to one’s spouse about better sun protection, and it was seen as fostering an understanding of why it is helpful for the relationship and spouse to engage in better sun protection. Participants felt Sun Safe Partners were easy to navigate, and their unique features were positively evaluated. These features may have increased partners’ engagement with the program and collaboration on homework, and made it more likely to impact relationship factors than the Generic Online intervention.

These conclusions should be considered in light of the study limitations. Data collection spanned the late summer through the winter months. For participants who resided in southern climates, the follow-up occurred in a warm, sunny time frame; however, for participants who resided in nonsouthern climates, follow-up occurred in the early fall when UV levels decreased. Thus, sun protection behaviors may not have been as useful among those residing in nonsouthern climates. Second, nearly 80% of the participants were non-Hispanic Whites. Thus, the sample size was not as diverse as the general population. However, skin cancer is far more prevalent among non-Hispanic Whites, especially those with highly sun-sensitive skin, so the sample undoubtedly contained a large number of high-risk participants, the key target population. Finally, our participants may have been more motivated to improve sun protection behaviors than the general population because they volunteered for an intervention on this topic.

This study leveraged a novel approach to facilitating engagement in sun protection by harnessing the relationship between spouses and cohabiting partners. A couple-focused intervention may hold promise as a way to improve sun protection behaviors by leveraging the concern, collaboration, and support among intimate partners and addressing relationship-based barriers to sun protection. Participants felt Sun Safe Partners Online was valuable, and most participants completed all of the modules. On the basis of the outcome of our pilot study, a fully powered trial with a larger, more diverse sample and a longer follow-up time frame is warranted to evaluate the efficacy of Sun Safe Partners Online, which has the potential in its web-based format to be scaled up to a larger population of adults at risk for skin cancer.

## Abbreviations

SPF: sun protection factor

## References

[1] (2018). Melanoma of the Skin. Cancer Statistics Center - American Cancer Society.

[2] (2019). Cancer Stat Facts: Melanoma of the Skin. Surveillance, Epidemiology, and End Results Program.

[3] Guy GP, Machlin SR, Ekwueme DU, Yabroff KR (2015). Prevalence and costs of skin cancer treatment in the US, 2002-2006 and 2007-2011. Am J Prev Med.

[4] US Department of Health and Human Services, Office of the Surgeon General (2019). The Surgeon General's Call to Action to Prevent Skin Cancer.

[5] van der Pols JC, Williams GM, Pandeya N, Logan V, Green AC (2006). Prolonged prevention of squamous cell carcinoma of the skin by regular sunscreen use. Cancer Epidemiol Biomarkers Prev.

[6] Green AC, Williams GM, Logan V, Strutton GM (2011). Reduced melanoma after regular sunscreen use: randomized trial follow-up. J Clin Oncol.

[7] (2019). What Are the Risk Factors for Skin Cancer?. Centers for Disease Control and Prevention.

[8] (2020). Skin Cancer Prevention. Skin Cancer Foundation.

[9] Fischer AH, Wang TS, Yenokyan G, Kang S, Chien AL (2016). Sunburn and sun-protective behaviors among adults with and without previous nonmelanoma skin cancer (NMSC): a population-based study. J Am Acad Dermatol.

[10] Buller DB, Cokkinides V, Hall HI, Hartman AM, Saraiya M, Miller E, Paddock L, Glanz K (2011). Prevalence of sunburn, sun protection, and indoor tanning behaviors among Americans: review from national surveys and case studies of 3 states. J Am Acad Dermatol.

[11] Bränström R, Kasparian NA, Chang Y, Affleck P, Tibben A, Aspinwall LG, Azizi E, Baron-Epel O, Battistuzzi L, Bergman W, Bruno W, Chan M, Cuellar F, Debniak T, Pjanova D, Ertmanski S, Figl A, Gonzalez M, Hayward NK, Hocevar M, Kanetsky PA, Leachman SA, Heisele O, Palmer J, Peric B, Puig S, Schadendorf D, Gruis NA, Newton-Bishop J, Brandberg Y (2010). Predictors of sun protection behaviors and severe sunburn in an international online study. Cancer Epidemiol Biomarkers Prev.

[12] Calderón TA, Bleakley A, Jordan AB, Lazovich D, Glanz K (2019). Correlates of sun protection behaviors in racially and ethnically diverse US adults. Prev Med Rep.

[13] Jackson KM, Aiken LS (2000). A psychosocial model of sun protection and sunbathing in young women: the impact of health beliefs, attitudes, norms, and self-efficacy for sun protection. Health Psychol.

[14] Weig EA, Tull R, Chung J, Brown-Joel ZO, Majee R, Ferguson NN (2020). Assessing factors affecting sunscreen use and barriers to compliance: a cross-sectional survey-based study. J Dermatolog Treat.

[15] Manne SL, Coups EJ, Kashy DA (2016). Relationship factors and couples' engagement in sun protection. Health Educ Res.

[16] Hay J, Ostroff J, Martin A, Serle N, Soma S, Mujumdar U, Berwick M (2005). Skin cancer risk discussions in melanoma-affected families. J Cancer Educ.

[17] Loescher LJ, Crist JD, Siaki LA (2009). Perceived intrafamily melanoma risk communication. Cancer Nurs.

[18] Prestwich A, Conner MT, Lawton RJ, Ward JK, Ayres K, McEachan RR (2014). Partner- and planning-based interventions to reduce fat consumption: randomized controlled trial. Br J Health Psychol.

[19] Voils CI, Coffman CJ, Yancy WS, Weinberger M, Jeffreys AS, Datta S, Kovac S, McKenzie J, Smith R, Bosworth HB (2013). A randomized controlled trial to evaluate the effectiveness of CouPLES: a spouse-assisted lifestyle change intervention to improve low-density lipoprotein cholesterol. Prev Med.

[20] Bowen DJ, Burke W, Hay JL, Meischke H, Harris JN (2015). Effects of web-based intervention on risk reduction behaviors in melanoma survivors. J Cancer Surviv.

[21] Butterfield RM, Lewis MA (2016). Health-related social influence: a social ecological perspective on tactic use. J Soc Pers Relatsh.

[22] Lewis MA, Butterfield RM, Darbes LA, Johnston-Brooks C (2016). The conceptualization and assessment of health-related social control. J Soc Pers Relatsh.

[23] Lewis MA, Gladstone E, Schmal S, Darbes LA (2006). Health-related social control and relationship interdependence among gay couples. Health Educ Res.

[24] Glanz K, Lewis FM, Rimer BK (1991). Health Behavior and Health Education. Med Sci Sports Exerc.

[25] Manne S, Day A, Coups EJ, Kashy D (2018). Sun safe partners: a pilot and feasibility trial of a couple-focused intervention to improve sun protection practices. Prev Med Rep.

[26] How to Select a Sunscreen. American Academy of Dermatology.

[27] Sun-Protective Clothing: A Safe, Simple Way to Keep the Rays at Bay. The Skin Cancer Foundation.

[28] Glanz K, Yaroch AL, Dancel M, Saraiya M, Crane LA, Buller DB, Manne S, O'Riordan DL, Heckman CJ, Hay J, Robinson JK (2008). Measures of sun exposure and sun protection practices for behavioral and epidemiologic research. Arch Dermatol.

[29] Hillhouse J, Turrisi R, Jaccard J, Robinson J (2012). Accuracy of self-reported sun exposure and sun protection behavior. Prev Sci.

[30] Glanz K, McCarty F, Nehl EJ, O'Riordan DL, Gies P, Bundy L, Locke AE, Hall DM (2009). Validity of self-reported sunscreen use by parents, children, and lifeguards. Am J Prev Med.

[31] O'Riordan DL, Nehl E, Gies P, Bundy L, Burgess K, Davis E, Glanz K (2009). Validity of covering-up sun-protection habits: association of observations and self-report. J Am Acad Dermatol.

[32] Janssen E, van Osch L, de Vries H, Lechner L (2011). Measuring risk perceptions of skin cancer: reliability and validity of different operationalizations. Br J Health Psychol.

[33] Manne S, Fasanella N, Connors J, Floyd B, Wang H, Lessin S (2004). Sun protection and skin surveillance practices among relatives of patients with malignant melanoma: prevalence and predictors. Prev Med.

[34] Manne SL, Coups EJ, Jacobsen PB, Ming M, Heckman CJ, Lessin S (2011). Sun protection and sunbathing practices among at-risk family members of patients with melanoma. BMC Public Health.

[35] Glanz K, Schoenfeld E, Weinstock MA, Layi G, Kidd J, Shigaki DM (2003). Development and reliability of a brief skin cancer risk assessment tool. Cancer Detect Prev.

[36] Kraemer HC, Mintz J, Noda A, Tinklenberg J, Yesavage JA (2006). Caution regarding the use of pilot studies to guide power calculations for study proposals. Arch Gen Psychiatry.

[37] Leon AC, Davis LL, Kraemer HC (2011). The role and interpretation of pilot studies in clinical research. J Psychiatr Res.

[38] Lewis MA, McBride CM, Pollak KI, Puleo E, Butterfield RM, Emmons KM (2006). Understanding health behavior change among couples: an interdependence and communal coping approach. Soc Sci Med.

